# The Value of Digital Rectal Examination (DRE) in Prostate Cancer Diagnostics

**DOI:** 10.7759/cureus.75390

**Published:** 2024-12-09

**Authors:** Adeoye Debo-Aina, Alexander Martindale, Juwayriyyah Amjad, Martina Smekal, Nkwam Nkwam

**Affiliations:** 1 Urology, Princess Royal University Hospital, King's College Hospital NHS Foundation Trust, London, GBR; 2 Urology, King's College Hospital NHS Foundation Trust, London, GBR

**Keywords:** dre, mri, pi-rads, prostate cancer, psa

## Abstract

Background and objective

Prostate cancer (PCA) is the most prevalent cancer among males. The National Institute for Health and Care Excellence (NICE) recommends referral to PCA diagnostic pathway based on two criteria: (1) abnormal digital rectal examination (DRE) and (2) elevated prostate-specific antigen (PSA). This study evaluates the diagnostic value of routine DRE in patients undergoing PCA assessment with pre-biopsy MRI.

Methods

We conducted a retrospective analysis of 436 patients on the PCA diagnostic pathway between September 2019 and June 2020, focusing on those with normal MRI [Prostate Imaging Reporting and Data System (PI-RADS) 1-2, n=147] and documented DRE results. Patients were categorised by their DRE status: normal vs. abnormal. The detection of prostate cancer and clinically significant prostate cancer (CSPC, i.e., Gleason score ≥7) was then compared between the two groups.

Results

The overall PCA detection rate was 10.2%, while it was 4.67% for CSPC. PCA and CSPC detection were higher with abnormal DRE (19.35% and 6.45% respectively) compared to normal DRE (7.76% and 4.31%). Among 23 patients biopsied, 65% (n=15) had PCA, with CSPC found in 30% (n=7). Of note, 61% (n=14) of biopsied patients had normal DRE, with nine PCA cases, five being CSPC, whereas 39% (n=9) with abnormal DRE had six PCA cases, two being CSPC. Statistical analysis using McNemar’s test showed no significant association between DRE and PCA diagnosis (p=0.146) or CSPC (p=0.774). Even though abnormal DRE was associated with higher PCA and CSPC detection rates, this finding was not statistically significant.

Conclusions

Based on our findings, PCA diagnostics can be effectively performed without DRE. This finding is pertinent when performing remote PCA diagnostic consultations, and it reevaluates DRE's value within the diagnostic pathway while emphasising a PSA- and MRI-based approach.

## Introduction

Prostate cancer (PCA) is the most common type of cancer among men. The National Institute for Health and Care Excellence (NICE) currently recommends referral to the PCA diagnostic pathway solely based on two criteria: (1) abnormal digital rectal examination (DRE) or (2) an elevated prostate-specific antigen (PSA) above the age-specific reference ranges [1]. As a result, DRE continues to be routinely performed as part of the clinical workup for patients clinically suspected to have PCA. Cancers of the prostate are commonly located in the peripheral zone and may be detected by DRE when tumor volumes exceed >0.2 cc [2].

The value of DRE in modern diagnostics for PCA has been a matter of debate. A 2003 meta-analysis comparing PSA and DREs as screening tools for PCA revealed DRE alone possesses a positive predictive value (PPV) of 17.8%, sensitivity of 53.2%, and specificity of 83.6%, whereas PSA has a PPV of 25.1%, sensitivity of 72.1%, and specificity of 93.2% in detecting PCA [3]. In contrast, an Irish retrospective study of patients undergoing transrectal ultrasound (TRUS) prostate biopsy highlighted the relevance of DRE in assessing for PCA, by revealing that DRE alone had an 81% sensitivity, 40% specificity, and 42% PPV [4]. The European Randomized Study of Screening for Prostate Cancer showed that the PPV of DRE in diagnosing PCA ranges between 4% and 33% in patients with PSA <3.0 ng/ml [5]. This increased to 49.6% with PSA >3.0 ng/ml, suggesting that the clinical benefit of DRE is dependent on the PSA level. Studies from the United States have shown that the absolute risk at 10 years for developing clinically significant prostate cancer (CSPC, i.e., Gleason score ≥7) is 23% with elevated PSA >3.0 ng/ml where suspicious DRE is present [6].

Investigations based only on PSA and DRE are associated with higher rates of negative biopsies, often diagnosing clinically insignificant disease, and are unable to accurately detect patients with clinically significant PCA [2]. Multiparametric MRI (mpMRI) is considered to be the most sensitive investigation in PCA diagnostics [7]. When used as a triage tool before biopsy, it has helped one in four men avoid unnecessary biopsies, while reducing the overdiagnosis of clinically non-significant prostate cancer by 5% and improving the detection of more clinically significant prostate cancers [8]. Patients referred with PSA above the age-specific reference range or with abnormal DRE, who are suspected to have localised prostate cancer and are suitable to have radical treatment, are offered MRI investigations [9].

The decision to proceed to biopsy is normally based on the MRI outcome of the likelihood of PCA detection, which is based on the Prostate Imaging-Reporting and Data System (PI-RADS) v2 or Likert scoring systems. Parameters such as serum PSA levels, PSA density (PSAD), and abnormal DRE findings are also considered in the decision-making process. Our local guidelines recommend evaluating MRIs using the PI-RADS. All patients with PI-RADS scores 3-5 are routinely offered biopsies. Whereas a PI-RADS score of 1 is a normal finding and these patients are not offered biopsies. PI-RADS 2 patients with a PSAD of >0.12 may also be offered biopsies, with consideration of other potential risk factors, such as abnormal DRE findings or significant family history of PCA. DRE has been reported to be an independent predictor of CSPC in patients with negative MRI [10]. This study aims to evaluate the diagnostic value of routine DRE in patients undergoing PCA assessment with pre-biopsy MRI.

## Materials and methods

Patients

This study involved a retrospective analysis of patients referred to the departmental Prostate Triage Assessment Clinic on a two-week wait referral as per the NICE guidelines for suspected prostate cancer. Data were collected retrospectively over 10 months between September 2019 and June 2020 from The Princess Royal University Hospital, in South East London. The data collected included patient age, DRE status, presenting PSA, PSA density (PSAD), PI-RADS scoring, decision to biopsy, and histological diagnosis (Gleason score). All data collected were anonymised and no individual patient-specific information was used.

Inclusion and exclusion criteria

Patients included in this study were prostate two-week wait referred, with normal MRI findings (PI-RADS 1-2), who had proceeded with prostate biopsies. These patients were classified according to DRE findings: (1) normal DRE and (2) abnormal DRE. Patients with no information documented regarding their DRE status were excluded. Patients with a known history of prostate cancer on active surveillance or watchful waiting were also excluded.

Analysis

Comparisons were made between patients with documented normal DRE and those with documented abnormal DRE concerning the overall prostate cancer detection, and detection of CSPC, defined as a Gleason score ≥7 (3+4 and 4+3). Statistical analysis for the comparison between the two patient groups was performed using McNemar’s test.

## Results

A total of 436 patients were reviewed on the prostate two-week wait pathway between September 2019 and June 2020. In this cohort, 192 patients had MRIs with a PI-RADS score of 1 (n=50) or 2 (n=142); 45 patients were excluded as they had no available documented DRE findings. Demographic characteristics of PI-RADS scores 1-2 patients are presented in Table 1. The overall PCA detection rate in patients with PI-RADS scores 1-2 (n=147) was 10.2%, with 4.67% for CSPC. The PCA detection rate in patients with normal recorded DRE (n=116) was 7.76%, with 4.31% for CSPC. Among those with recorded abnormal DRE (n=31), 19.35% were diagnosed with PCA and 6.45% with CSPC; 23 patients (n=1, PI-RADS score 1; n=22, PI-RADS score 2) among 147 patients with documented DRE with a PI-RADS score of 1 or 2 proceeded to have a prostate biopsy.

**Table 1:** Demographics of PCA diagnostics in patients with normal MRI (PI-RADS scores 1-2) CSPC: clinically significant prostate cancer; DRE: digital rectal examination; IQR: interquartile range; MRI: magnetic resonance imaging; PCA: prostate cancer; PI-RADS: Prostate Imaging Reporting and Data System; PSA: prostate-specific antigen

Variables	Normal DRE	Abnormal DRE	Total
Number of patients	116	31	147
Age, years, median (IQR)	65 (30-86)	66 (46-77)	65 (30-86)
PSA, ng/mL, median (IQR)	6.6 (0.3-29)	6.4 (0.7-16.5)	6.6 (0.3-29)
PSA density, ng/ml^2^, median (IQR)	0.1 1 (0.01-0.85)	0.08 (0.02-0.29)	0.1 (0.01-0.85)
Prostate cancer, n (%)	9 (7.76%)	6 (19.35%)	15 (10.2%)
CSPC, n (%)	5 (4.31%)	2 (6.45%)	7 (4.67%

**Figure 1:**
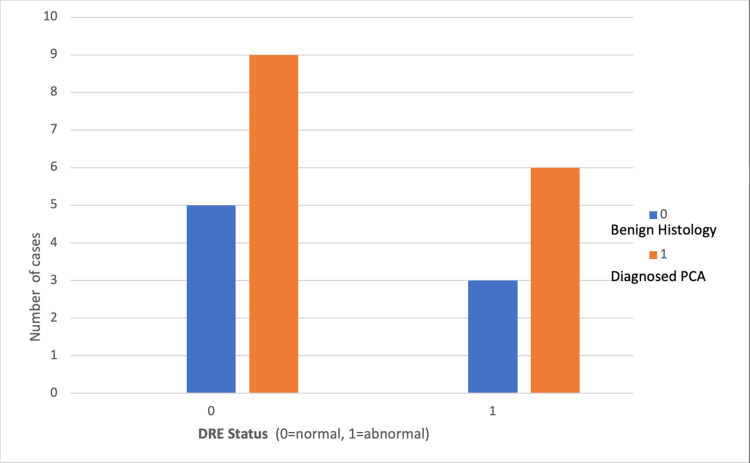
Diagnosed PCA cases and DRE status in PI-RADS 1-2 patients following prostate biopsy DRE: digital rectal examination; PCA: prostate cancer; PI-RADS: Prostate Imaging Reporting and Data System

**Figure 2:**
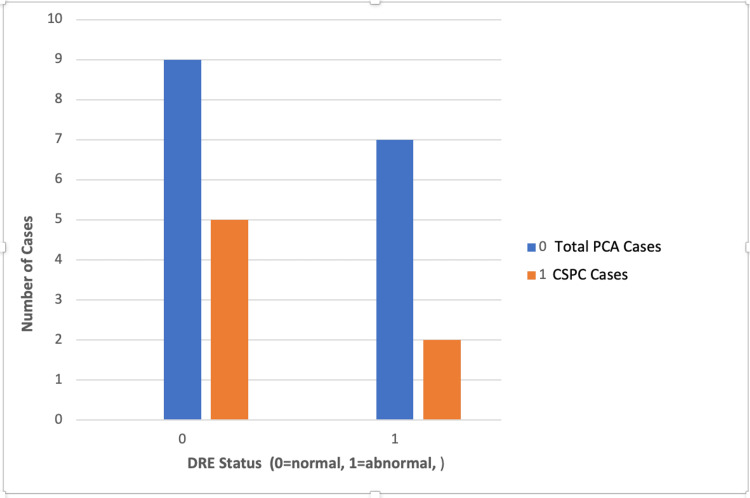
CSPC cases and DRE status in PI-RADS 1-2 patients CSPC: clinically significant prostate cancer; DRE: digital rectal examination; PCA: prostate cancer; PI-RADS: Prostate Imaging Reporting and Data System

Of note, 65% (n=15) of PI-RADS 1-2 patients who had a prostate biopsy with a documented DRE finding had confirmed prostate cancer on histology (Figure 1). These patients all had a PI-RADS score of 2. Notably, the only PI-RADS 1 patient with confirmed abnormal DRE and biopsy had benign histology. CSPC, defined as a Gleason score ≥7 (3+4 and 4+3), was found in 30% (n=7) of the cohort; 61% of biopsied PI-RADS 1-2 patients had a documented confirmed benign feeling prostate on DRE (n=14), with nine confirmed cases of prostate cancer found in this group, five being CSPC. In the remaining 39% (n=9) with documented abnormal prostates on DRE, six cases of prostate cancer were confirmed with only two being CSPC (Figure 2).

McNemar’s test was used to determine if there was any significant difference between patient groups with documented normal vs. abnormal DRE findings. No significant association was found between DRE findings (benign or abnormal) and prostate cancer diagnosis on histology in PI-RADS 1-2 patients (p=0.146). Nor was there an association between DRE findings and diagnosing clinically significant prostate cancer within this group (p=0.774).

In light of these findings, for patients with low radiological suspicion of prostate cancer (PI-RADS score 1-2), DRE findings are not significantly associated with histologically confirmed prostate cancer diagnosis. However, a large proportion of PI-RADS 1-2 patients who underwent biopsy had prostate cancer. This is likely due to the selection of patients with high clinical risk factors, which were not explored in this analysis (e.g., PSA, family history).

## Discussion

DRE was historically once considered the sole and primary modality of assessing the prostate for evidence of malignant disease, before the advent of PSA testing. Its simplicity and cost-effective nature provide clinicians with insights into the size, consistency, nodularity, and asymmetry of the prostate. However, this, in turn, is heavily influenced by the judgement and experience of the performing clinician [11]. The coronavirus 2019 (COVID-19) pandemic led to significant changes to the structure and format of the delivery of the National Health Service (NHS) in the United Kingdom, through broader implementation of technology, with face-to-face appointments being replaced with virtual consultations either by telephone or video. This restructuring resulted in many PCA investigative pathways relying on PSA and MRI without the need for a DRE. Furthermore, the increasing use of MRI in the referral pathway has made the role of an isolated DRE even more uncertain [12], leading to debates about the contributory value of DRE in modern PCA diagnostics.

One prospective study examining the role of DRE both as an adjunctive and investigative tool in prostate cancer diagnosis revealed that DRE had an overall diagnostic accuracy in suspected prostate cancer patients of 63.45% (sensitivity: 59.51%, specificity: 67.23%, and PPV: 63.53%). This was only applicable in cases of PI-RADS score ≥3 on MRI, and PSA >4 ng/ml. This study further identified a positive correlation between a positive DRE and the recognition of prostate cancer within the peripheral zone, as well as a positive association between a positive DRE and clinically significant disease with Gleason scores ≥7. It did, however, highlight the limited value of DRE in combination with MRI, showing that it provided no additional diagnostic value when used with MRI [13].

Our study showed no significant correlation between DRE status and the overall diagnosis of PCA, or clinically significant PCA in PI-RADS 1-2 patients, suggesting that DRE holds limited diagnostic value in patients with normal MRI findings (PI-RADS scores 1-2). In our study, 19.35% of patients with abnormal DRE with PI-RADS scores 1-2 were found to have PCA compared to 7.76% with normal DRE. This can be explained by the smaller number of patients with abnormal DRE. PI-RADS score 2 is considered a normal MRI finding, reflecting benign prostatic hyperplasia (BPH) changes only. It has been reported that there is no significant detection of CSPC in PI-RADS 2 patients [14]. Many local PCA diagnostics protocols do not even routinely offer prostate biopsies to patients with these MRI findings, irrespective of PSAD. However, there is also data to suggest that approximately 10% of significant lesions are still nonvisible on MRI [15], and hence prostate biopsies do play an important role, even in PI-RADS 2, and our local guidelines are guided by PSAD.

DRE usage remains a controversial subject within the prostate cancer referral and diagnostic pathway. Although an abnormal DRE is associated with an increased likelihood of having CSPC, it has little clinical value in patients with normal PSA. The relative and absolute risk of having CSPC is significantly much higher in men with abnormal DRE with elevated PSA ≥3 ng/ml. As such. the United States’ National Comprehensive Cancer Network (NCCN) advises that DRE would serve better as an adjunct for follow-up in patients with elevated PSA and advocates for referrals based on PSA testing within primary care, thereby avoiding unnecessary referrals based on DRE [6].

In a 2018 [16] systematic review and meta-analysis, DRE was found to have poor performance in PCA screening within primary care. It was recommended not to be used as a screening tool to avoid risks of unnecessary testing, overdiagnosis, and treatment. A 2022 study [17] identified a poor level of concordance between DREs performed within primary vs. secondary care following referral - around 46% - with the rate of CSPC detection being 16% and 14% within primary and secondary care respectively. A 3% CSPC detection rate was seen in cases where an abnormal DRE was found in the presence of a normal PSA within primary care. However, when PSA was elevated with a concurrently abnormal DRE, the CSPC detection rate increased to 29%. Furthermore, they reveal the benefit of using PSA to reduce the number of repeated DREs within secondary care (with a 30% savings on repeat DRE), while helping to triage men with abnormal DRE directly to MRI.

Our study has a few limitations. Our focus was confined to the detection of PCA and CSPC in patients with radiological presumed normal MRIs (PI-RADS scores 1 and 2). We did not investigate the correlation between DRE and prostate cancer diagnosis in patients with PI-RADS score ≥3, for which DRE has been shown to have a better positive predictive value [13]. Only 23 patients with PI-RADS scores 1-2 on MRI and documented DRE status underwent prostate biopsies. This constitutes a small sample size and may have potentially influenced the results of this study and led to a lack of statistical power in our analysis.

Although our results show a limited role for DRE in PCA diagnosis, it is still considered essential in the diagnostic investigations for PCA, especially in helping to identify low-PSA and high-grade PCAs, which are often aggressive and neuroendocrine in nature [18]. Hence, it is useful to investigate the diagnostic outcomes of patients referred with normal-low PSA who have abnormal DRE in a future study. As such it would still be a good clinical practice for patients to undergo further investigations following MRI, and be physically examined. In our department, it is common practice to conduct physical examinations, including a DRE for all males who undergo prostate biopsy as part of their pre-procedural assessment. Lastly, a biparametric MRI (bpMRI) is the modality of radiological investigations for PCA in our department. It is associated with reduced scanning time, is very cost-effective at a population level, and allows for greater access to MRI. However, there is still uncertainty as to whether bpMRI has similar detection rates for PCA in comparison to standard mpMRI [19].

## Conclusions

Our data does not endorse the hypothesis that an abnormal DRE finding leads to a higher detection rate of prostate cancer. We recommend that PCA diagnostics can be safely undertaken remotely, without requiring face-to-face consultations for DRE, if biopsies are routinely offered in PI-RADS 2 cases with PSAD >0.12. This is especially relevant in the post-COVID-19 era, where there has been a global shift towards remote consultation.
